# Discontinuation and tapering of prescribed opioids and risk of overdose among people on long-term opioid therapy for pain with and without opioid use disorder in British Columbia, Canada: A retrospective cohort study

**DOI:** 10.1371/journal.pmed.1004123

**Published:** 2022-12-01

**Authors:** Mary Clare Kennedy, Alexis Crabtree, Seonaid Nolan, Wing Yin Mok, Zishan Cui, Mei Chong, Amanda Slaunwhite, Lianping Ti

**Affiliations:** 1 British Columbia Centre on Substance Use, Vancouver, British Columbia, Canada; 2 School of Social Work, University of British Columbia–Okanagan, Kelowna, British Columbia, Canada; 3 School of Population and Public Health, University of British Columbia, Vancouver, British Columbia, Canada; 4 Department of Medicine, University of British Columbia, St. Paul’s Hospital, Vancouver, British Columbia, Canada; 5 British Columbia Centre for Disease Control, Vancouver, British Columbia, Canada; UNSW Sydney, AUSTRALIA

## Abstract

**Background:**

The overdose crisis in North America has prompted system-level efforts to restrict opioid prescribing for chronic pain. However, little is known about how discontinuing or tapering prescribed opioids for chronic pain shapes overdose risk, including possible differential effects among people with and without concurrent opioid use disorder (OUD). We examined associations between discontinuation and tapering of prescribed opioids and risk of overdose among people on long-term opioid therapy for pain, stratified by diagnosed OUD and prescribed opioid agonist therapy (OAT) status.

**Methods and findings:**

For this retrospective cohort study, we used a 20% random sample of residents in the provincial health insurance client roster in British Columbia (BC), Canada, contained in the BC Provincial Overdose Cohort. The study sample included persons aged 14 to 74 years on long-term opioid therapy for pain (≥90 days with ≥90% of days on therapy) between October 2014 and June 2018 (*n* = 14,037). At baseline, 7,256 (51.7%) persons were female, the median age was 55 years (quartile 1–3: 47–63), 227 (1.6%) persons had been diagnosed with OUD (in the past 3 years) and recently (i.e., in the past 90 days) been prescribed OAT, and 483 (3.4%) had been diagnosed with OUD but not recently prescribed OAT. The median follow-up duration per person was 3.7 years (quartile 1–3: 2.6–4.0). Marginal structural Cox regression with inverse probability of treatment weighting (IPTW) was used to estimate the effect of prescribed opioid treatment for pain status (discontinuation versus tapered therapy versus continued therapy [reference]) on risk of overdose (fatal or nonfatal), stratified by the following groups: people without diagnosed OUD, people with diagnosed OUD receiving OAT, and people with diagnosed OUD not receiving OAT. In marginal structural models with IPTW adjusted for a range of demographic, prescription, comorbidity, and social-structural exposures, discontinuing opioids (i.e., ≥7-day gap[s] in therapy) was associated with increased overdose risk among people without OUD (adjusted hazard ratio [AHR] = 1.44; 95% confidence interval [CI] 1.12, 1.83; *p* = 0.004), people with OUD not receiving OAT (AHR = 3.18; 95% CI 1.87, 5.40; *p* < 0.001), and people with OUD receiving OAT (AHR = 2.52; 95% CI 1.68, 3.78; *p* < 0.001). Opioid tapering (i.e., ≥2 sequential decreases of ≥5% in average daily morphine milligram equivalents) was associated with decreased overdose risk among people with OUD not receiving OAT (AHR = 0.31; 95% CI 0.14, 0.67; *p* = 0.003). The main study limitations are that the outcome measure did not capture overdose events that did not result in a healthcare encounter or death, medication dispensation may not reflect medication adherence, residual confounding may have influenced findings, and findings may not be generalizable to persons on opioid therapy in other settings.

**Conclusions:**

Discontinuing prescribed opioids was associated with increased overdose risk, particularly among people with OUD. Prescribed opioid tapering was associated with reduced overdose risk among people with OUD not receiving OAT. These findings highlight the need to avoid abrupt discontinuation of opioids for pain. Enhanced guidance is needed to support prescribers in implementing opioid therapy tapering strategies with consideration of OUD and OAT status.

## Abstract 2

In a retrospective cohort study from Canada, Dr Mary Kennedy and colleagues explore the effect of discontinuation and tapering of prescribed opioids on risk of overdose among people on long-term opioid therapy for pain with and without opioid use disorder.

## Author summary

**Why was this study done?:**

- In Canada and the United States, a rise in opioid-related morbidity and mortality has prompted system-level efforts to restrict opioid prescribing for chronic pain.
- Guidelines implemented in Canada and the United States have recommended tapering prescribed opioids for pain to the lowest effective dose, potentially discontinuing therapy, among people receiving opioid therapy for chronic pain when risks outweigh benefits.
- Although there is emerging evidence to suggest that deprescribing opioid therapy for chronic pain may increase risk of opioid-related harms, most existing studies have focused on non-representative subpopulations.
- To our knowledge, no studies to date have examined whether the effects of discontinuing and tapering opioid therapy for pain on overdose risk might differ among people with and without concurrent opioid use disorder.

**What did the researchers do and find?:**

- Drawing on administrative data linked at the individual level for a random sample of residents registered in the provincial health insurance client roster in British Columbia, Canada, we identified 14,037 persons prescribed long-term opioid therapy for pain between October 2014 and June 2018.
- We examined associations between discontinuation and tapering of prescribed opioid therapy for pain (versus continued treatment) and risk of overdose, stratified by whether patients had been diagnosed with opioid use disorder and recently prescribed opioid agonist therapy.
- We found that discontinuing opioid therapy for pain was associated with increased overdose risk among people without opioid use disorder (adjusted hazard ratio [AHR] = 1.44; 95% confidence interval [CI] 1.12, 1.83; *p* = 0.004). However, stronger associations were observed among people with opioid use disorder, including those not receiving opioid agonist therapy (AHR = 3.18; 95% CI 1.87, 5.40; *p* < 0.001) and those receiving opioid agonist therapy (AHR = 2.52; 95% CI 1.68, 3.78; *p* < 0.001).
- Tapering opioid therapy for pain was associated with decreased overdose risk among people with opioid use disorder not receiving opioid agonist therapy (AHR = 0.31; 95% CI 0.14, 0.67; *p* = 0.003).

**What do these findings mean?:**

- Abrupt discontinuation of prescribed opioid treatment for pain is contraindicated given its association with increased risk of overdose.
- Enhanced guidance is needed to support healthcare providers in implementing safe and effective strategies for tapering opioid treatment for pain that are tailored to the unique needs of individual patients, with particular consideration of opioid use disorder and prescribed opioid agonist therapy status.

## Introduction

Canada and the United States are in the midst of an ongoing overdose crisis, and opioid-related overdose is now a leading cause of accidental death in both countries [1,2]. The province of British Columbia (BC), Canada, is among the jurisdictions that have been most severely impacted by the overdose crisis [3]. In April 2016, the BC government declared a public health emergency in response to a rapid rise in the number of overdose deaths in the province [4]. Since then, there have been more than 10,000 illicit drug toxicity deaths in BC, including more than 2,260 deaths in 2021 alone, yielding an annual mortality rate of 43.6 per 100,000 population, a rate more than double that recorded in 2016 (20.4 per 100,000 population) [5].

The most recent and pronounced wave of the overdose crisis has been driven in large part by the proliferation of illicitly manufactured fentanyl and fentanyl analogues in unregulated drug markets [6,7]. However, the initial rise in overdose deaths in North America in the 2000s stemmed largely from increased dispensing of prescribed opioids for pain [6,7]. Additionally, over the past decade, growing evidence has demonstrated associations between opioid prescribing characteristics, including high-dose and long-term prescribing, and increased risk of harms such as opioid use disorder (OUD) and overdose death [8,9]. Furthermore, the magnitude of treatment effects of opioids for chronic non-cancer pain have been found to be modest at best [10,11].

In light of this evidence and increased awareness of the role of prescribed opioid dispensing as a driver of the initial iteration of the overdose crisis, a range of system-level interventions to restrict opioid prescribing for pain have been implemented in Canada and the United States in recent years [12–14]. For example, following the declaration of the overdose crisis as a public health emergency in BC, the College of Physicians and Surgeons of British Columbia released guidelines for prescribing opioids for chronic non-cancer pain in June 2016 [12]. In May 2017, the National Pain Centre released Canadian guidelines [13]. Among other recommendations, both guidelines encouraged gradual tapering of prescribed opioids to the lowest effective dose, potentially discontinuing treatment, among patients receiving high-dose opioid therapy for chronic non-cancer pain when risks outweigh benefits [12,13].

While the aforementioned initiatives have been implemented with the intention of curtailing unsafe prescribing and related harms, there is emerging evidence to suggest that deprescribing opioid therapy may produce unintended negative consequences [15–22]. Of note, several studies have suggested that discontinuation of opioid therapy for pain may increase risk of illicit opioid use [20,21] and harms such as overdose death and overdose-related healthcare encounters [16,17,19]. Additionally, a recent retrospective cohort study of patients on long-term, high-dose opioid therapy for pain found that the 1-year period following dose tapering was associated with increased risk of overdose and mental health crisis [18]. Further, a follow-up study found that the increased risk of these adverse events extended up to 2 years after taper initiation [23]. However, the limited research to date investigating the link between deprescribing opioids for pain and opioid-related harms has some important shortcomings, with most existing studies focusing on subpopulations that may not be representative of the broader population of people prescribed opioids for pain [16,18,19,23]. Further, these studies have primarily relied on standard regression techniques to control for confounding [16,18,19], which can produce biased effect estimates when time-dependent confounding factors that are also affected by opioid treatment status (e.g., dose) are present [24].

It is also noteworthy that, to our knowledge, no studies to date have examined whether the potential impacts of tapering and discontinuing prescribed opioids for pain on opioid-related outcomes may differ between people with and without concurrent OUD. This is a notable evidence gap given that people with OUD might be more likely to use unregulated opioids when experiencing barriers in accessing prescribed opioids [25], which could exacerbate the risk of harms such as overdose. Conversely, a large body of research has demonstrated the benefits of opioid agonist therapy (OAT) in reducing overdose risk in this subpopulation [26,27], and therefore receipt of OAT could plausibly modify the risk of overdose among people with OUD whose opioid treatment for pain is discontinued or tapered. Therefore, we undertook the present study to examine associations between discontinuing and tapering prescribed opioids for pain and risk of overdose among a representative sample of people on long-term opioid therapy for pain in BC, stratified by diagnosed OUD and prescribed OAT status.

## Methods

### Data sources and study design

We conducted a retrospective population-based cohort study of people on long-term opioid therapy for pain in BC. The cohort was constructed using data from a 20% random sample of residents registered in the provincial health insurance client roster in BC which is contained within the BC Provincial Overdose Cohort [28]. The 20% random sample of residents in the client roster was generated in SQL using the standard Oracle random number generator (DBMS_RANDOM) [29]. Registration in the client roster provides access to provincial health insurance and is mandatory for all residents of BC, including Canadian citizens, permanent residents, people on visas >6 months, and dependents of people in these categories residing in BC [28]. As part of the response to the overdose public health emergency declared in BC [4], the BC Provincial Overdose Cohort was developed and linked to a range of province-wide administrative data sources, including death, coroner, drug and poison control, emergency department, and ambulance surveillance records (1 January 2015–31 December 2018), as well as hospital, primary care, community pharmacy prescription dispensing, corrections, and social assistance records (1 January 2010–31 December 2018) [28]. These records were linked at the individual level using a series of determinist and probabilistic algorithms that incorporate the name, birthdate, and personal health number assigned to each person in the client roster [28].

Further information on datasets in the cohort is provided in S1 Text. The study analysis plan is provided in S2 Text. A consultation with the University of British Columbia research ethics board confirmed that ethics approval and informed consent were not required for this study as it was conducted as part of the BC Centre for Disease Control’s core public health functions.

### Study population

**Fig 1:**
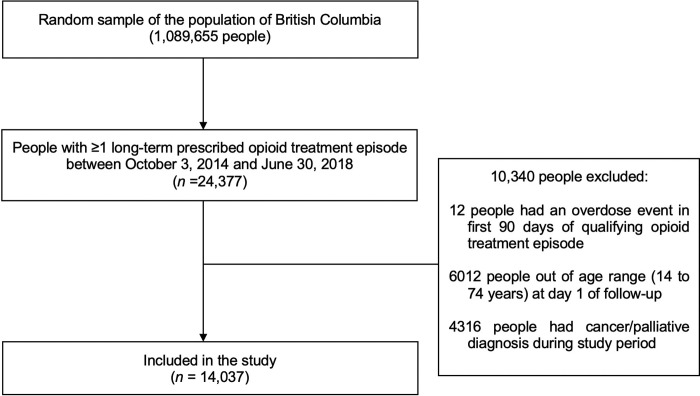
Flowchart showing how the analytic sample (*n* = 14,037) was determined.

For the present study, we restricted the cohort to persons who had ≥1 long-term episode of being prescribed opioid treatment for pain (≥90 days, with ≥90% of days on therapy) between 3 October 2014 and 30 June 2018. Thus, the earliest follow-up start date (day 1 after the first 90 days of an eligible treatment episode) was 1 January 2015 (to coincide with the start date when data from all data sources to assess overdose were available). Consistent with past work [30], opioid treatment for pain was based on community pharmacy prescription dispensing records, and included all opioids not prescribed for treatment of OUD (as identified through pharmacy fee codes) except for low-dose opioids for cough suppression. A full list of opioid medications included in this definition is available in S1 Text. Consistent with previous studies using data from the cohort [30,31], we also restricted the study sample to persons aged ≥14 and ≤74 years to include the most common age demographic of persons experiencing overdose in BC [5]. We further restricted the cohort to persons who did not have primary or acute care codes for treatment of cancer or palliative care during the study period, and who did not experience an overdose (as defined below) between day 1 and 90 of the index episode of opioid treatment for pain. Additionally, all persons included in the study had to be present in the client roster database for ≥3 years prior to their follow-up start date. Fig 1 presents a flow chart showing how the analytic sample was selected. Follow-up was censored at 31 December 2018 or date of death, whichever occurred first.

### Measures

The primary outcome was overdose, which included both nonfatal and fatal overdose events [28]. Nonfatal overdose events were identified through drug and poison control, ambulance, emergency department, hospital, and physician billing records, while fatal overdose events were identified through coroner and death records [28]. The data-source-specific case definitions of nonfatal and fatal overdose are provided in S1 Text. Healthcare records <24 hours apart were collapsed into a single overdose event to reduce the potential for overcounting of events.

The primary exposure variable was prescribed opioid treatment for pain status, which was a time-updated 3-level variable (discontinuation, tapered therapy, or continued therapy [reference category]) that was measured using community pharmacy prescription dispensing records, including drug identification numbers (DINs), and records of start dates, quantity dispensed, days’ supply, and dose of prescriptions for opioids for pain. Continued and discontinued therapy were defined as <7-day and ≥7-day gaps, respectively, in therapy following the date at which the supply of a prescription would have run out if taken every day. Consistent with Canadian guidelines for opioids for chronic non-cancer pain [12,13], tapering therapy was defined as ≥2 sequential relative decreases of ≥5% in average daily morphine milligram equivalents (MME), where such dose decreases were separated by ≤42 days. Thus, an overall ≥10% decrease in average daily MME was required to be defined as a tapering event. Beginning on the follow-up start date, treatment status on each day of follow-up for each participant was defined as 1 of the aforementioned 3 categories (i.e., discontinued, tapered, or continued therapy). There was no upper limit for the duration of periods defined as discontinued therapy. When persons met the criteria for tapered treatment, they began contributing person-time towards tapering events at the time of the first decrease in average daily MME, and ceased contributing person-time towards tapering events once they met one of the pre-specified criteria for ending a tapering event (i.e., discontinued treatment, dose increase, or stable dose) as detailed in S1 Text. Further details regarding measurement of the prescribed opioid treatment for pain status variable are provided in S1 Text.

The effect modifier of interest was a 3-level measure of diagnosed OUD and prescribed OAT status (no OUD diagnosis; OUD diagnosis but not recently prescribed OAT; OUD diagnosis and recently prescribed OAT). Consistent with past studies [32,33], OUD diagnosis was defined as having 2 primary care visits or 1 hospitalization for OUD in a given year in the past 3 years, or having been prescribed OAT (including methadone, buprenorphine/naloxone, and/or slow-release oral morphine) in the past 3 years. Prescribed OAT status, which was defined as having been prescribed OAT for OUD in the past 90 days, was measured using community pharmacy records, including product identification numbers (PINs) specifically allocated when the aforementioned OAT medications are prescribed as treatment for OUD, and records of start dates, quantity dispensed, and days’ supply. We should note that we had initially defined OAT prescription as prescription in the past 3 years. However, we redefined this as a past-90-day measure as we agreed with a reviewer’s assessment that a shorter time frame would be preferable given our interest in understanding how more recent OAT prescription might modify the associations of interest. The diagnosed OUD and prescribed OAT variable was treated as time-updated. See S1 Text for more information.

We considered a range of variables as potential confounders, including calendar year (2018, 2017, 2016, or 2015), sex at baseline (female or male), age (per year increase), and regional health authority (Northern, Fraser, Vancouver Coastal, Vancouver Island, or Interior). We also considered prescription-related variables as confounders; these variables were based on community pharmacy prescribing dispensation records (including DINs/PINs and records of start dates, quantity dispensed, days’ supply, and dose of prescribed medications) and included the following: average daily MME (≥200, 90–199, 50–89, or ≥0–49), type of prescribed opioid for pain (long-acting versus short-acting versus tramadol only) [16], benzodiazepine/z-drug use (yes versus no), other sedating medication use (yes versus no), non-sedating antidepressant use (yes versus no), and non-sedating antipsychotic use (yes versus no). All prescribing variables referred to dispensed medications in the previous 90 days unless otherwise indicated. We also considered several comorbidity and drug-related variables as potential confounders, which were measured based on the past 3 years using primary and acute care data and included the following: Elixhauser index score (without mental health conditions) (≥2, 1, or 0) [30,34], respiratory comorbidities (yes versus no), cardiovascular comorbidities (yes versus no), mental health conditions (more severe, less severe, or none), and injection drug use (yes versus no) [35]. Finally, we considered institutionalization variables of hospitalization (yes versus no) and incarceration (yes versus no), which referred to the past 30 days, as potential confounders. All explanatory variables were treated as time-updated unless otherwise indicated. Further details regarding measurement of confounding variables are provided in S1 Text.

### Statistical analysis

First, we compared the baseline characteristics of the study sample, stratified by OUD and OAT status, using the Kruskal–Wallis test for continuous variables, and Pearson’s chi-squared test or Fisher’s exact test (when expected cell counts were ≤5) for categorical variables. Next, we fit 3 separate bivariable Cox regression models for recurrent events (one for each of level of the diagnosed OUD and prescribed OAT status variable) to estimate the unadjusted association between prescribed opioid treatment for pain status (i.e., discontinued, tapered, or continued therapy [reference]) and risk of overdose. To estimate the effect of prescribed opioid treatment for pain status on risk of overdose, stratified by OUD and OAT status, we used marginal structural Cox models with inverse probability of treatment weighting (IPTW) [24,36], fitting a separate model for each of the 3 levels of the diagnosed OUD and prescribed OAT status variable. This method can account for time-varying variables that are simultaneously confounders of the effect of interest and affected by previous treatment status (e.g., average daily MME), and can also adjust for selection bias caused by non-random treatment assignment [24,36]. We calculated stabilized weights to reduce variability due to instability in estimation that can be produced by dominant subjects [24].

As a supplementary analysis requested by a reviewer, we reran the aforementioned marginal structural Cox regression analyses but as a series of models that were increasingly adjusted for groups of confounders, starting with demographic variables (age; sex; regional health authority; calendar year), followed by prescription and drug-related variables (average daily MME; prescribed opioid type; injection drug use; benzodiazepine/z-drug use; other sedating medication use; non-sedating antidepressant use; non-sedating antipsychotic use), then comorbidity variables (Elixhauser index score [without mental health conditions]; respiratory comorbidities; cardiovascular comorbidities; mental health conditions), and finally institutionalization variables (hospitalization; incarceration). Additionally, as a sensitivity analysis requested by a reviewer, we reran the bivariable and marginal structural Cox regression analyses using an alternative measure of the exposure of interest (prescribed opioid treatment for pain status), where continued and discontinued therapy were defined as <14-day and ≥14-day gaps, respectively, in therapy following the date at which the supply of a prescription would have run out if taken every day. All analyses were performed using SAS Enterprise Guide 7.1. All *p*-values are 2-sided. The study is reported in accordance with the Strengthening the Reporting of Observational Studies in Epidemiology (STROBE) guidelines for cohort studies (see S1 STROBE Checklist).

## Results

A total of 14,037 persons on long-term opioid therapy for pain were included in the present study and were followed for a median duration of 3.7 years (quartile 1–3: 2.6–4.0), collectively contributing a total of 44,756.5 person-years of observation. At baseline, 7,256 (51.7%) persons were female, the median age was 55 years (quartile 1–3: 47–63), and 9,871 (70.3%) had an average daily prescribed opioid for pain dose of ≥0–49 MME in the previous 90 days. A total of 13,327 (94.9%) persons had not been diagnosed with OUD at baseline, 227 (1.6%) had been diagnosed with OUD and recently prescribed OAT, and 483 (3.4%) had been diagnosed with OUD but not recently prescribed OAT. Table 1 reports the baseline characteristics of the sample stratified by diagnosed OUD and prescribed OAT status at baseline. Table A in S3 Text presents the transition rate (per 100 person-years) between levels of diagnosed OUD and prescribed OAT status among the study sample during follow-up.

**Table 1:** Baseline characteristics among people on long-term opioid therapy for pain in British Columbia, Canada, stratified by OUD* and OAT^†^ status (*n* = 14,037).

Characteristic	No diagnosed OUD* n = 13,327	Diagnosed OUD* but not prescribed OAT^†^ *n* = 483	Diagnosed OUD* and prescribed OAT^†^ *n* = 227	*p* Value
**Age (per year older)**				
Median (Q1–Q3)	56 (48–63)	50 (40–57)	48 (40–57)	<0.001
**Sex**				
Female	6,934 (52.0)	240 (49.7)	82 (36.1)	<0.001
Male	6,393 (48.0)	243 (50.3)	145 (63.9)	
**Regional health authority**				
Interior	3,240 (24.3)	111 (23.0)	32 (14.1)	<0.001
Fraser	4,118 (30.9)	126 (26.1)	66 (29.1)	
Vancouver Coastal	1,804 (13.5)	112 (23.2)	82 (36.1)	
Vancouver Island	2,903 (21.8)	103 (21.3)	42 (18.5)	
Northern	1,262 (9.5)	31 (6.4)	≤5 (2.2)	
**Year of follow-up start date**				
2015	9,421 (70.7)	388 (80.3)	156 (68.7)	<0.001
2016	1,950 (14.6)	56 (11.6)	34 (15.0)	
2017	1,334 (10.0)	29 (6.0)	27 (11.9)	
2018	622 (4.7)	10 (2.1)	10 (4.4)	
**Average daily MME** ^†^				
≥0 to 49	9,610 (72.1)	161 (33.3)	100 (44.1)	<0.001
50 to 89	1,636 (12.3)	63 (13.0)	22 (9.7)	
90 to 199	1,258 (9.4)	119 (24.6)	46 (20.3)	
≥200	823 (6.2)	140 (29.0)	59 (26.0)	
**Prescribed opioid type** ^†^				
Long-acting	3,379 (25.4)	278 (57.6)	123 (54.2)	<0.001
Short-acting	8,337 (62.6)	196 (40.6)	103 (45.4)	
Tramadol only	1,611 (12.1)	9 (1.9)	≤5 (0.4)	
Elixhauser index score* ^**#**^				
0	2,828 (21.2)	16 (3.3)	9 (4.0)	<0.001
1	3,718 (27.9)	76 (15.7)	46 (20.3)	
≥2	6,718 (50.9)	391 (81.0)	172 (75.8)	
Respiratory comorbidities*				
Yes	859 (6.5)	41 (8.5)	22 (9.7)	0.033
No	12,468 (93.5)	442 (91.5)	205 (90.3)	
Cardiovascular comorbidities*				
Yes	1,480 (11.1)	61 (12.6)	13 (5.7)	0.020
No	11,847 (88.9)	422 (87.3)	214 (94.2)	
Mental health conditions*				
More severe	1,492 (11.2)	389 (80.5)	203 (89.4)	<0.001
Less severe	201 (1.5)	18 (3.7)	≤5 (0.4)	
None	11,634 (87.3)	76 (15.7)	23 (10.1)	
Injection drug use*				
Yes	389 (2.9)	32 (68.7)	90 (39.7)	<0.001
No	12,938 (97.1)	151 (31.3)	137 (60.3)	
**Benzodiazepine/z-drug use** ^†^				
Yes	4,835 (36.3)	229 (47.4)	91 (40.1)	<0.001
No	8,492 (63.7)	254 (52.6)	136 (59.9)	
**Other sedating medication use** ^†^				
Yes	246 (1.9)	25 (5.2)	6 (2.6)	<0.001
No	13,081 (98.1)	458 (94.8)	221 (97.4)	
**Non-sedating antidepressant use** ^†^				
Yes	4,169 (31.3)	196 (40.6)	67 (29.5)	0.001
No	9,158 (68.7)	287 (59.4)	160 (70.5)	
**Non-sedating antipsychotic use** ^†^				
Yes	171 (1.3)	14 (2.9)	7 (3.1)	0.001
No	13,156 (98.7)	469 (97.1)	220 (96.9)	
**Hospitalization** ^‡^				
Yes	494 (3.7)	25 (5.2)	≤5 (1.8)	0.071
No	12,833 (96.3)	458 (94.8)	223 (98.2)	
**Incarceration** ^‡^				
Yes	14 (0.1)	≤5 (0.2)	≤5 (2.2)	<0.001
No	13,313 (99.9)	482 (99.8)	222 (97.8)	

Data are *n* (%) unless otherwise indicated.

*In the 3 years prior to baseline.

^†^In the 90 days prior to baseline.

^#^Elixhauser index score without mental health conditions.

^‡^In the 30 days prior to baseline.

MME, morphine milligram equivalents; OAT, opioid agonist therapy; OUD, opioid use disorder.

A total of 530 (3.8%) persons experienced at least 1 overdose event during follow-up, with a total of 827 overdose events among the study sample, yielding an incidence density rate of 1.85 events per 100 person-years (95% confidence interval [CI] 1.73, 1.98). This included 429 (3.1%) persons who experienced at least 1 nonfatal overdose event (707 events total) and 120 (0.9%) who experienced a fatal overdose event. Stratified by OUD and OAT status, this included 420 overdose events (nonfatal and fatal) among those who had not been diagnosed with OUD (incidence density rate = 1.0 events per 100 person-years; 95% CI 0.9, 1.1), 296 events among those diagnosed with OUD and prescribed OAT (incidence density rate = 14.7 events per 100 person-years; 95% CI 13.1, 16.5), and 111 events among those diagnosed with OUD but not prescribed OAT (incidence density rate = 9.9 events per 100 person-years; 95% CI 8.2, 11.9).

A total of 12,812 (91.3%) persons experienced at least 1 prescribed opioid treatment for pain discontinuation event during follow-up, with a total of 83,113 discontinuation events among the study sample. Stratified by OUD and OAT status, this included 78,776 discontinuation events among those who had not been diagnosed with OUD (median days per event = 31 [quartile 1–3: 16–60]), 2,659 events among those diagnosed with OUD and prescribed OAT (median days per event = 18 [quartile 1–3: 7–42]), and 1,678 events among those diagnosed with OUD but not prescribed OAT (median days per event = 26 [quartile 1–3: 11–53]).

A total of 9,861 (70.3%) persons experienced at least 1 prescribed opioid treatment for pain tapering event during follow-up, with a total of 42,181 tapering events among the study sample. Stratified by OUD and OAT status, this included 39,015 tapering events among those who had not been diagnosed with OUD (median days per event = 57 [quartile 1–3: 24–127]), 1,191 events among those diagnosed with OUD and prescribed OAT (median days per event = 43 [quartile 1–3: 16–99]), and 1,975 events among those diagnosed with OUD but not prescribed OAT (median days per event = 43 [quartile 1–3: 17–99]).

Table 2 presents the unadjusted and adjusted estimates of the effects of discontinuation and tapering of opioid treatment for pain on risk of overdose, stratified by OUD and OAT status. As shown, in marginal structural Cox models using IPTW, discontinuing opioid treatment for pain was associated with increased risk of overdose among people who had not been diagnosed with OUD (adjusted hazard ratio [AHR] = 1.44; 95% CI 1.12, 1.83), people with diagnosed OUD who had received OAT (AHR = 2.52; 95% CI 1.68, 3.78), and people with diagnosed OUD who had not received OAT (AHR = 3.18; 95% CI 1.87, 5.40). Tapering opioid treatment for pain was associated with decreased risk of overdose among people with OUD who had not received OAT (AHR = 0.31; 95% CI 0.14, 0.67) in marginal structural Cox models using IPTW.

**Table 2:** Regression analyses of the effects of discontinuation and tapering of prescribed opioid treatment for pain on risk of overdose among people on long-term opioid therapy for pain in British Columbia, Canada, stratified by diagnosed OUD and prescribed OAT status.

Group	Unadjusted estimates		Adjusted estimates*	
	Hazard ratio (95% CI)	*p* Value	Hazard ratio (95% CI)	*p* Value
**No diagnosed OUD** ^†^				
Continued prescribed opioid treatment	Ref		Ref	
Tapered prescribed opioid treatment	1.59 (1.19, 2.13)	0.002	1.14 (0.84, 1.53)	0.404
Discontinued prescribed opioid treatment	1.24 (0.98, 1.57)	0.079	1.44 (1.12, 1.83)	0.004
**Diagnosed OUD**^†^ **but not prescribed OAT**^‡^				
Continued prescribed opioid treatment	Ref		Ref	
Tapered prescribed opioid treatment	0.47 (0.22, 1.01)	0.052	0.31 (0.14, 0.67)	0.003
Discontinued prescribed opioid treatment	2.34 (1.36, 4.04)	0.002	3.18 (1.87, 5.40)	<0.001
**Diagnosed OUD**^†^ **and prescribed OAT**^‡^				
Continued prescribed opioid treatment	Ref		Ref	
Tapered prescribed opioid treatment	0.75 (0.39, 1.47)	0.402	0.61 (0.30, 1.22)	0.163
Discontinued prescribed opioid treatment	0.95 (0.63, 1.42)	0.783	2.52 (1.68, 3.78)	<0.001

*Using marginal structural modeling with inverse probability of treatment weights; models adjusted for the following: calendar year, sex, age, regional health authority, average daily morphine milligram equivalents, type of prescribed opioid, Elixhauser index score (without mental health conditions), respiratory comorbidities, cardiovascular comorbidities, mental health conditions, injection drug use, benzodiazepine/z-drug use, other sedating medication use, non-sedating antidepressant use, non-sedating antipsychotic use, hospitalization, and incarceration.

^†^In the past 3 years.

^‡^In the past 90 days.

CI, confidence interval; OAT, opioid agonist therapy; OUD, opioid use disorder.

Table B in S3 Text presents the results of the marginal structural Cox regression analyses that were increasingly adjusted for groups of confounders. As shown, demographic confounders appeared to play the most important role in shifting estimates of the associations of interest. Table C in S3 Text presents the results of the sensitivity analysis using an alternative measure of prescribed opioid treatment for pain status, where continued and discontinued therapy were defined as <14-day and ≥14-day gaps, respectively, in therapy. As shown, adjusted estimates of the associations of interest remained largely unchanged from the Cox analyses using the primary measure of the exposure.

## Discussion

In this population-based study of people prescribed long-term opioid therapy for pain, we found that approximately 4% of the study sample experienced at least 1 nonfatal or fatal overdose event over an average of approximately 4 years of follow-up. In marginal structural models, compared to continued treatment, discontinuation of opioid treatment for pain was associated with increased risk of overdose regardless of diagnosed OUD and prescribed OAT status. However, this association was stronger among people with diagnosed OUD. Among people with diagnosed OUD who had not been recently prescribed OAT, tapering of opioid treatment for pain was associated with decreased risk of overdose compared to continued treatment.

To our knowledge, this study is the first to estimate associations between discontinuation and tapering of opioid treatment for pain and risk of overdose, stratified by diagnosed OUD and prescribed OAT status, among a representative sample of people prescribed long-term opioid therapy for pain. Our finding of an association between discontinuation of opioid therapy and increased risk of overdose is consistent with several previous studies demonstrating positive associations between discontinued opioid treatment for pain and risk of adverse opioid-related events, including overdose death and overdose-related healthcare encounters [16,17,19]. However, our study builds on past research in demonstrating that the association between opioid treatment discontinuation and overdose risk was more pronounced among those with diagnosed OUD. The heightened overdose risk associated with opioid therapy discontinuation may be explained by people transitioning to using alternative non-prescribed and/or unregulated sources of opioids to manage pain, withdrawal, and other symptoms after treatment discontinuation [20,21]. In particular, although we were unable to determine the source of substances involved in all overdose events examined herein, individuals may have been more likely to use substances containing illicitly manufactured fentanyl, which has increasingly been involved in overdose events in BC in recent years [5,37,38], including more than 73% of overdose deaths that occurred in the province during the study period [5,37]. Moreover, after a period of therapy discontinuation, people may have reduced physiological tolerance for opioids, which could further exacerbate overdose risk [39–41]. Persons with diagnosed OUD could be especially likely to use non-prescribed and/or unregulated opioids to alleviate symptoms after opioid treatment discontinuation [25], which may explain the stronger association between treatment discontinuation and overdose risk in this group that was observed in this study. An alternative explanation for the stronger treatment discontinuation association observed among those with OUD is the higher average daily MME among people in this group compared to those without OUD. Although the stratified marginal structural Cox models were adjusted for recent average daily MME, persons with OUD may have experienced comparatively larger absolute dose reductions with therapy discontinuation than those without OUD as a result of their higher prior doses, which could partly account for the stronger discontinuation association for this group.

Although we expected to observe an attenuated association between therapy discontinuation and overdose risk among persons with OUD prescribed OAT compared to those with OUD not prescribed OAT, our findings suggest that association estimates did not significantly differ between these 2 subgroups. The explanations for this finding are unclear. However, this might be because OAT prescription is a marker of greater OUD severity [42], which could have masked the benefits of OAT in attenuating overdose risk stemming from therapy discontinuation. We may also have neglected to control for important confounders (e.g., use of unregulated drugs; socioeconomic status) in the adjusted models because it was not possible to assess these using the available data. It is also possible that our analysis was underpowered to detect differential associations due to small numbers of persons with OUD prescribed OAT. Further research may help to elucidate potential explanations for our finding of a lack of differential discontinuation association estimates based on OAT prescription status among people with OUD.

We also observed a protective association between tapered opioid treatment for pain and overdose risk among people with diagnosed OUD who were not prescribed OAT, and found no evidence of an association between tapering and overdose risk among people without diagnosed OUD or among people with diagnosed OUD who had been prescribed OAT. These findings stand in contrast with those of a previous study by Agnoli et al. that documented an association between periods following opioid tapering and increased risk of overdose among patients on long-term, stable high-dose opioid therapy for pain [18]. These incongruent findings might be explained in part by methodological differences between studies. In particular, the Agnoli et al. study was restricted to patients prescribed higher-dose (≥50 MME) therapy [18], whereas approximately 70% of our sample had a baseline dose <50 MME. Given that absolute decreases in dose translate into larger relative dose decreases at lower versus higher baseline dose levels, and that we employed a lower minimum dose reduction threshold (≥10% decrease in mean dose versus ≥15% in Agnoli et al.), our tapering measure would have been comparatively more sensitive in capturing smaller dose reductions, which may be less likely to pose risks [22]. It is also noteworthy that our finding of a lack of significant association between opioid tapering and overdose risk among people without concurrent OUD and people with OUD prescribed OAT aligns with other studies suggesting a lack of adverse impacts related to opioid tapering among people with chronic pain, particularly when tapering is gradual and accompanied by additional supports [43–45].

There are several possible explanations for our finding that tapering was protective against overdose risk only among people with diagnosed OUD who were not recently prescribed OAT. First, opioid tapering could be an indicator of patient stabilization and positive clinical outcomes among this subpopulation [43]. Additionally, a diagnosis of OUD may result in patients receiving more intensive clinician support and monitoring while undergoing opioid tapering, and being connected to other services and supports (e.g., take-home naloxone; care from clinicians trained in addiction medicine), which could help to mitigate the risk of harms [12,46]. As for the non-significant association between tapering and overdose risk for those with OUD who had recently been prescribed OAT, this finding might be explained by OAT attenuating the protective association between tapering and overdose risk that was observed among other people with OUD. Specifically, given the established benefits of OAT in providing protection against overdose [27], tapering of opioids for pain may not confer additional benefits in reducing overdose risk in persons receiving OAT. Alternatively, the non-significant tapering association among the subgroup prescribed OAT might be explained in part by OAT prescription being an indicator of greater OUD severity [42], which could have obscured potential beneficial effects of tapering in reducing overdose risk. Our analyses may also have been underpowered to detect a potential tapering association for the subgroup of people with OUD prescribed OAT because of the small sample size. Further research is needed to better understand potential underlying explanations for the differential associations between tapering and overdose risk by OUD and OAT status observed in this study.

Our findings have implications for policy and practice decisions concerning opioid prescribing for chronic pain. Specifically, our findings underscore the need for healthcare providers and policymakers to carefully consider potential unintended adverse effects of discontinuing opioid treatment for chronic pain when developing prescribing interventions and making practice decisions [16,20]. Given the harms of opioid treatment discontinuation identified in this and past studies, non-consensual and abrupt discontinuation of opioid treatment for pain is contraindicated in almost all instances [16,17,19–21,46]. Further, our findings reinforce calls for enhanced guidance to support healthcare providers in implementing safe and effective opioid tapering strategies that are tailored to the unique needs of individual patients [16,46,47]. In particular, guidelines and policies should support prescribers in identifying patients with concurrent OUD and modifying treatment plans accordingly, as our findings suggest that engaging this subpopulation in tapering might be beneficial in terms of mitigating the risk of overdose if OAT has not recently been prescribed, while discontinuing therapy may be especially likely to increase the risk of overdose among people with OUD. Effective tapering strategies that may reduce risks while supporting positive clinical outcomes could include engaging patients in shared and consensual decision-making regarding treatment plans, implementing slower tapering protocols, providing close monitoring during tapering, and facilitating access to pharmacological and non-pharmacological therapies to manage pain and OUD, as appropriate [16,18,21,46–48]. Future studies should further examine the effectiveness of such approaches among chronic pain patients with and without concurrent OUD.

This study has several limitations. First, our outcome measure did not capture overdose events that did not involve a healthcare encounter or result in death. Further, we were unable to determine the source of drugs involved in overdose events (i.e., whether these were illicitly sourced and/or prescribed) as this information was not recorded in all of the datasets used to derive the outcome measure. Additionally, our measurement of opioid treatment status was based on dispensation of medications and may not reflect actual adherence to therapy. It is also possible that the pharmacy fee codes used to define opioids as prescribed for treatment of pain may have been used by some clinicians when actually prescribing opioids off label to treat OUD. Another limitation is that, although we employed only one measure of opioid tapering, there is considerable variability in how tapering has been operationalized in the existing literature [18,49,50]. We were also unable to assess circumstances surrounding opioid tapering or discontinuation. Additionally, although we note above that the differential associations observed in this study may be partly explained by the higher average dose of prescribed opioids among people with OUD compared to those without OUD, our sample size was not large enough to examine possible effect modification by further stratifying our models on the basis of dose category. Future studies should seek to examine the potential role of opioid dose in modifying the associations observed herein. We also did not distinguish between instances when OAT prescription reflected initiation versus continuation of therapy in our measurement of the effect modifier of interest, and thus future studies should seek to assess how this factor might modify associations between discontinuing and tapering opioid therapy for pain and overdose risk. A further limitation is that we were unable to control for some notable confounders in our analyses, including use of unregulated drugs and socioeconomic status [51,52], because these were not available in the data. Another possible limitation is the risk of immortal time bias, particularly given that persons needed to survive longer to be assigned to the tapered category of the exposure of interest than to the other two categories, which may have resulted in biased estimates of association. Finally, although our study is based on data from a random population-based sample of persons on long-term opioid therapy in BC, our findings may not be generalizable to persons on long-term opioid therapy in other settings in Canada or elsewhere.

This study found that, compared to continued treatment, discontinuing opioid treatment among people on long-term therapy for pain was associated with increased risk of overdose, particularly among people with diagnosed OUD. Tapering opioid treatment for pain was associated with reduced risk of overdose among people with diagnosed OUD who had not recently been prescribed OAT. These findings point to the need to avoid abrupt discontinuation of opioid treatment for pain and to enhance guidance for prescribers in modifying opioid treatment tapering strategies on the basis of OUD and OAT status.

## Supporting information

S1 STROBE Checklist (DOCX)

S1 Text Description of datasets and variables. (DOCX)

S2 Text Study analysis plan. (DOC)

S3 Text Supplementary analyses. (DOCX)

## Abbreviations

AHR: adjusted hazard ratio
BC: British Columbia
CI: confidence interval
DIN: drug identification number
IPTW: inverse probability of treatment weighting
MME: morphine milligram equivalents
OAT: opioid agonist therapy
OUD: opioid use disorder
PIN: product identification number
