# Correction: Discontinuation and tapering of prescribed opioids and risk of overdose among people on long-term opioid therapy for pain with and without opioid use disorder in British Columbia, Canada: A retrospective cohort study

**DOI:** 10.1371/journal.pmed.1004213

**Published:** 2023-03-21

**Authors:** The PLOS Medicine Staff

## Notice of Republication

This article was republished on March 1, 2023, to provide the correct Academic Editor name. Please download this article again to view the correct version.
