# Supplementary material for: Discontinuation and tapering of prescribed opioids and risk of overdose among people on long-term opioid therapy for pain with and without opioid use disorder in British Columbia, Canada: A retrospective cohort study
Source: PLoS Med. 2022 Dec 1;19(12):e1004123. doi: 10.1371/journal.pmed.1004123 (PMC9714711; doi:10.1371/journal.pmed.1004123)
Supplement: S1 Text — (DOCX) [file pmed.1004123.s002.docx]

**S1 Text: Description of datasets and variables**

Administrative datasets

1. **Ministry of Health Provincial Client Roster.** A register of BC residents (Canadian citizens, permanent residents, people on visas longer than 6 months, and dependents of people in these categories residing in BC). Registration with the client roster is mandatory for BC residents and provides those registered with access to provincial health insurance (1).
2. **BC Emergency Health Services (BCEHS).** Records information about the time, nature and location of overdose events attended by ambulance. Events coded as overdose by the attending paramedics.
3. **Drug and Poison Information Centre (DPIC).** Records details about calls to the provincial poison hotline by the public or medical personnel for clinical advice on poisoning management.
4. **BC Coroner’s Service (BCCS).** Records details on all accidental and undetermined illicit drug toxicity deaths in BC, as all of these deaths are referred to the coroner for investigation (2).
5. **BC Vital Statistics.** Records demographic and medical information on deaths (3).
6. **Enhanced Emergency Department (EED) records.** Case-based reporting of opioid-related overdoses treated in emergency departments in three of five BC Health Authorities.
7. **National Ambulatory Care Reporting System (NACRS).** Records on ambulatory care within BC, covering 67% of ED visits in the province and almost 100% of emergency department visits in the two urban health authorities (4).
8. **Discharge Abstract Database (DAD).** Records of all stays in acute care hospitals in BC (5).
9. **Medical Services Plan (MSP).** Records all fee-for-service provider (e.g., primary care) visits billed to BC’s health insurance program (6).
10. **BC Corrections.** Records of all admissions and discharges from BC correctional facilities for people aged 18 years and older. This does not include incarceration records for federal correctional facilities (7).
11. **Social assistance.** Records of all social assistance payments were provided by the Ministry of Social Development and Poverty Reduction (MSDPR). There are multiple programs that provide monetary assistance to people who are experiencing, for example, unemployment or underemployment, disability, food insecurity, or housing instability (8,9).
12. **PharmaNet (PNET).** Records of all provincial community pharmacy dispensations in BC (10).

**Primary outcome, exposure and effect modifier variable definitions**

Outcome: Overdose

| **Dataset** | **Overdose definition** |
| --- | --- |
| British Columbia Emergency Health Services (BCEHS) | Naloxone administered by paramedics, or impression code for: recreational drug overdose AND cardiac or respiratory arrest/death, overdose/poisoning (ingestion), sick, unconscious, or impression code for: opioid related, opioid related / OD or cardiac arrest AND overdose/poisoning (ingestion) |
| Drug and Poison Information Centre (DPIC) | AAPCCGenCode indicative of opioids (37701–05, 37707–8, 37784, 41700, 72700, 72702, 72704, 77810, 200625, 200628, 200630, 200638, 201063, 201131) |
| Enhanced Emergency Department (EED) | Where the physician assessed the clinical symptoms as indicating an opioid overdose |
| Medical Services Plan (MSP) | Visits with an international classification of disease (ICD) 9th edition ICD-9) and 10th edition (ICD-10) codes of 965.0, 965.00, 965.01, 965.02, 965.09, E8500 |
| Discharge Abstract Database (DAD) | Primary discharge diagnosis ICD-10 code of T40.0, T40.1, T40.3, T40.4, T40.6 |
| National Ambulatory Care Reporting System (NACRS) | Records with an ICD-10 code of T40.0, T40.1, T40.3, T40.4, T40.6 ED discharge diagnosis field |
| British Columbia Coroner’s Service (BCCS) | All deaths recorded as accidental and undetermined illicit drug toxicity deaths referred to the coroner for investigation |
| BC Vital Statistics | ICD-10 code of T40.0, T40.1, T40.3, T40.4, T40.6 |

**Exposure: Prescribed opioid for pain treatment status**

**Medications included in opioid class**

| **Medication group** | **Medications included** | **Dataset** |
| --- | --- | --- |
| Opioids | Anileridine, buprenorphine (excluding for opioid use disorder), butorphanol, codeine, diamorphine, fentanyl, hydrocodone, hydromorphone, levorphanol, meperidine, methadone (excluding for opioid use disorder), morphine (excluding for opioid use disorder), oxycodone, oxymorphone, pentazocine, propoxyphene, tapentadol, tramadol | PharmaNet (Drug Identification Numbers (DIN)/ Product Identification Numbers (PIN)) |

**Summary of prescribed opioid for pain treatment status measure:**

Three-level measure, based on prescribed opioid dispensations:

(1) Continued therapy (<7-day gap(s) in therapy (following the date at which the supply of a prescription would have run out if taken every day));

(2) Discontinued (≥7-day gaps in therapy (following the date at which the supply of a prescription would have run out if taken every day);

(3) Tapering (≥2 sequential relative decreases of ≥5% in average daily morphine milligram equivalents (ME), where such dose decreases were separated by ≤42 days).

Detailed definition of prescribed opioid for pain treatment status measure:

Stage 1:

**(A)** Identified opioid therapy treatment episodes (defined based on a group of one or more dispensations with <182-day gap from the last day of supply of the previous dispensation to the next dispensation date) (11,12). That is, episodes were separated by at least 182 days. Therefore, an individual could have multiple episodes during their follow-up period if their follow-up period was long enough to have multiple episodes. Episodes could be either acute or long-term. However, only long-term episodes (i.e., ≥90 days with ≥90% of days on therapy) were used for tapering assessment.

**(B)** Each episode of being on opioid therapy was partitioned every 14 days into a tapering period (TP) until the end of the episode.

**(C)** The average daily ME for each TP was calculated. Average daily ME was defined as total daily ME/total days on therapy in each TP (i.e., the maximum denominator would be 14 days).

**(D)** The relative *change* in average daily ME between consecutive TPs was calculated to categorize remaining TPs into continued, discontinued or tapering (as defined below). If a gap between **2 consecutive tapering TPs** was ≥14 days, then tapering was further divided into 2 separate tapering episodes.

**Tapering definition (for Stage 1)**

When the following three criteria were met, tapering started (i.e., persons began contributing person-time to a tapering event) at the beginning of TP(t) with the first decrease in average daily ME and continued until tapering ended:

- - 1. There were at least two relative decreases of ≥5% in average daily ME from one TP to a subsequent TP AND
  - 2. Each dose decrease was separated by ≤3 TPs (i.e., 42 days) AND
  - 3. The average dose in the TP after the second decrease was < the average dose between the two decreases < the average dose in the TP before the first decrease.

**Note:** Individuals were defined as tapering even if the decreases of ≥5% in average daily ME were not in consecutive TPs but each decrease occurred within 42 days (i.e., ≤3 TPs).

- For stage 1, tapering ended (i.e., persons stopped contributing person-time to a tapering event) at the last day on therapy in TP(t) if one of the following scenarios was met:
  - There was a break in treatment of ≥14 days from the last day of treatment in TP(t) and the beginning of treatment in TP(t+1).
  - There was no subsequent decrease of ≥5% in average daily ME for >3TPs.
  - TP(t+2) ≥ TP(t+1) > TP(t) (i.e., average daily ME continued to increase for 2 consecutive TPs or increase for immediately next TP and then remained stable in next TP after that).
  - TP(t+1) > TP(t+2) > TP(t) (i.e., increase in average daily ME but then decreased to a dose that is still higher than the dose before it was increased).

**Stage 2** (to categorize each day within an episode as continued, tapering or discontinued therapy)**:**

For each continued or tapering TP (i.e., non-discontinued TPs) defined in Stage 1, if there was a ≥7-day gap in treatment within one of these TPs or between 2 consecutive non-discontinued TPs, this gap was categorized as discontinued. The rest of the days within a single TP retained the same TP categorization. If a <7-day gap lied between a ‘tapering’ TP and a ‘continued’ TP, then that gap was categorized as the treatment status prior to the gap. That is, if the ‘continued’ TP occurred before the ‘tapering’ TP then the gap between these statuses was categorized as continued. However, if the ‘tapering’ TP occurred before the ‘continued’ TP, the gap was categorized as tapering.

**Effect modifier: Opioid use disorder (OUD) and opioid agonist therapy (OAT) status**

OUD and OAT status was a three-level measure, and treated as time-updated:

1. No OUD diagnosis in the past 3 years;
2. OUD diagnosis in the past 3 years but not prescribed OAT in the past 90 days;
3. OUD diagnosis in the past 3 years and prescribed OAT in the past 90 days.

Definitions of OUD diagnosis and OAT are as follows:

| **Diagnosis** | **ICD9 codes** | **ICD10 codes** | **Definition** | **Datasets** |
| --- | --- | --- | --- | --- |
| Opioid use disorder | 304.00 – 304.03  304.70 – 304.73 305.50-305.53 | F11 | Either 2 primary care visits or 1 hospitalization for OUD in a given year in the past 3 years | MSP, DAD |

**Opioid use disorder**

**Medications included in OAT category**

| **Medication group** | **Medications included** | **Dataset** |
| --- | --- | --- |
| Opioid agonist therapy | Buprenorphine (for opioid use disorder), methadone (for opioid use disorder), or slow-release oral morphine (for opioid use disorder) prescribed at least once in the past 90 days | PharmaNet (DIN/PIN)* |

***Note:** DIN/PIN for OAT are specifically allocated for OUD treatment (as opposed to the treatment of pain).

**Confounding variable definitions**

**Prescribed medication variables**

| **Variable** | **Comparisons** | **Dataset** | **Medications included** |
| --- | --- | --- | --- |
| Average daily ME‡ | 0-49 ME/day; 50-89 ME/day; 90-199 ME/day; 200+ ME/day. | PharmaNet (DIN/PIN) | Anileridine, buprenorphine (excluding for opioid use disorder), butorphanol, codeine, diamorphine, fentanyl, hydrocodone, hydromorphone, levorphanol, meperidine, methadone (excluding for opioid use disorder), morphine (excluding for opioid use disorder), oxycodone, oxymorphone, pentazocine, propoxyphene, tapentadol, tramadol |
| Type of opioid treatment ‡# | Long-acting opioid, short-acting opioid vs. tramadol only | PharmaNet (DIN/PIN) | Anileridine, buprenorphine (excluding for opioid use disorder), butorphanol, codeine, diamorphine, fentanyl, hydrocodone, hydromorphone, levorphanol, meperidine, methadone (excluding for opioid use disorder), morphine (excluding for opioid use disorder), oxycodone, oxymorphone, pentazocine, propoxyphene, tapentadol, tramadol |
| Use of benzodiazepines/z-drugs‡ | Yes *vs.* No | PharmaNet (DIN/PIN) | Alprazolam, bromazepam, chlordiazepoxide, clobazam  clonazepam, clorazepate, diazepam  estazolam, flurazepam, ketazolam  lorazepam, nitrazepam, oxazepam  temazepam, triazolam, zaleplon  zolpidem, zopiclone |
| Use of other sedating medications‡ | Yes *vs.* No | PharmaNet (DIN/PIN) | Amitriptyline, amoxapine, clomipramine, desipramine, doxepin, imipramine, maprotiline, mirtazapine, nefazodone, nortriptyline, phenelzine, protriptyline,tranylcypromine, trazodone, trimipramine, tryptophan, asenapine, butaperazine, chlorpromazine, chlorprothixene, clozapine, flupentixol, fluspirilene, loxapine, mesoridazine, methotrimeprazine, olanzapine, periciazine, piperacetazine, pipotiazine, prochlorperazine, promazine, quetiapine,  remoxipride, risperidone, thiopropazate, thioproperazine, thioridazine, ziprasidone, zuclopenthixol, gabapentin,  pregabalin, carbamazepine, lamotrigine, levetiracetam, phenytoin, topiramate, valproic acid, baclofen, cyclobenzaprine, methocarbamol, choral hydrate, phenobarbital, nabilone |
| Use of non-sedating antidepressants‡ | Yes *vs.* No | PharmaNet (DIN/PIN) | Bupropion, citalopram, desvenlafaxine, duloxetine, escitalopram, fluoxetine,  Fluvoxamine, isocarboxazid, levomilnacipran, moclobemide, nomifensine, paroxetine, sertraline, venlafaxine, vilazodone, vortioxetine |
| Use of non-sedating antipsychotics‡ | Yes *vs.* No | PharmaNet (DIN/PIN) | Aripiprazole, fluphenazine, haloperidol, lurasidone, paliperidone, perphenazine, pimozide, tiotixene, trifluoperazine |
| **Notes:**  ‡ Refers to ≥1 dispensation of a medication in the previous 90 days.  # Refers to opioid prescribed in the previous 90 days with a hierarchal classification as follows: long-acting opioid, short-acting opioid, or tramadol only (13). For example, if an individual was prescribed both short- and long-acting opioids, that individual would be defined as having been prescribed long-acting opioids. | | | |

**Comorbidity and institutionalization variables**

| **Variable** | **Comparisons** | **ICD9 codes** | **ICD10 codes** | **Definition** | **Datasets** |
| --- | --- | --- | --- | --- | --- |
| Respiratory comorbidities*# | Yes *vs.* No | **Asthma:** 493  **Chronic obstructive pulmonary disease:** 491, 492, 496 | **Asthma:** J45  **Chronic obstructive pulmonary disease:** J41, J42, J43, J44 | Either 2 primary care visits or 1 hospitalization in a given year in the past 3 years | MSP, DAD |
| Cardiovascular comorbidities*† | Yes *vs.* No | **Ischemic heart disease:** 410-414  **Heart failure:** 428  **Hypertension:** 401, 402, 403, 404, 405  **Stroke:** 362.3, 430, 431, 433, 434, 436 | **Ischemic heart disease:** I20-I25  **Heart failure:** I50  **Hypertension:** I10, I11, I12, I13, I15  **Stroke:** H34.1, I60, I61, I63 (exclude I63.6), I64 | Either 2 primary care visits or 1 hospitalization in a given year in the past 3 years | MSP, DAD |
| Mental health Conditions*‡ | More severe, less severe vs. no mental health condition | **More severe:**  Schizophrenia (295, 297, 298);  Bipolar disorder (296);  Personality disorder (301).  **Less severe:**  Depression (300.4; 311; 50B (also requires another code to qualify));  Anxiety (300 (excluding 300.4); 50B (also required another code to qualify));  Stress/adjustment disorders (308; 309). | **More severe:**  Schizophrenia (F20, F21, F22, F23, F24, F25, F28, F29);  Bipolar disorder (F30, F31, F34 (excluding F34.1), F38, F39);  Personality disorder (F60, F61, F69).  **Less severe:**  Depression (F32, F33, F34.1);  Anxiety (F40, F41));  Stress/adjustment disorders (F43). | Either 2 primary care visits or 1 hospitalization in a given year in the past 3 years | MSP, DAD |
| Hospitalization** | Yes *vs.* No | N/A | N/A | At least once in the past 30 days | DAD |
| Incarceration** | Yes *vs.* No | N/A | N/A | At least once in the past 30 days | BCCS |
| **Notes:**  *Refers to previous 3 years.  # Includes asthma and/or chronic obstructive pulmonary disease.  † Includes ischemic heart disease, heart failure, hypertension, and/or stroke.  ‡ As defined in Smolina et al., 2020 (14), with the following 3 categories: (1) no mental health condition; (2) typically less severe mental health conditions (depression, anxiety and/or stress/adjustment disorders); (3) typically more severe mental health conditions (schizophrenia, bipolar and/or personality disorders). For example, if an individual meets the definition of having both a ‘less severe’ and ‘more severe’ mental health condition, that individual would be defined as having a more severe mental health condition.  ** Refers to previous 30 days. | | | | | |

**Injection drug use** (yes vs. no) is based on validated algorithm using MSP and DAD data and refers to either 2 primary care visits or 1 hospitalization for injectables in the past 3 years. This variable has been described in detail in Janjua et al., 2018 (15).

**Elixhauser comorbidity index score** (≥2, 1 vs. 0) was derived from DAD and was modified by excluding

ICD-10 codes for mental health conditions given that these were assessed separately (14,16).

**References**

1. MacDougall L, Smolina K, Otterstatter M, Zhao B, Chong M, Godfrey D, et al. Development and characteristics of the Provincial Overdose Cohort in British Columbia, Canada. Lima VD, editor. PLoS ONE. 2019 Jan 10;14(1):e0210129.

2. BC Coroners Service. Illicit drug toxicity deaths in BC (January 1, 2011 - January 31, 2021). Burnaby, BC: Office of the Chief Coroner; 2022 [cited 2022 Feb 22]. Available from: https://www2.gov.bc.ca/assets/gov/birth-adoption-death-marriage-and-divorce/deaths/coroners-service/statistical/illicit-drug.pdf

3. BC Vital Statistics Agency. BC Vital Statistics Deaths: Data Extract. Victoria, BC: Ministry of Health; 2021. Available from: https://www2.gov.bc.ca/gov/content/life-events

4. BC Ministry of Health. National Ambulatory Care Reporting System (NACRS): Data Extract. Vancouver: Data Stewardship Committee; 2017.

5. Canadian Institute of Health Information [creator]. Discharge Abstract Database (Hospital Separations). BC Ministry of Health [publisher]. Data Extract. MOH; 2017.

6. BC Ministry of Health. Medical Services Plan (MSP) Payment Information File: Data Extract. Vancouver: Data Stewardship Committee; 2017.

7. Ministry of Public Safety and the Solicitor General. A profile of BC corrections. Vancouver: Ministry of Public Safety and the Solicitor General; 2017.

8. Ministry of Social Development and Poverty Reduction. Ministry of Social Development and Poverty Reduction. 2021. Available from: https://www2.gov.bc.ca/gov/content/governments/organizational-structure/ministries-organizations/ministries/social-development-poverty-reduction.

9. Government of British Columbia. Together BC: British Columbia’s Poverty Reduction Strategy. BC: Government of British Columbia; 2019.

10. BC Ministry of Health. PharmaNet (PNet): Data Extract. Vancouver: Data Stewardship Committee; 2017.

11. Smolina K, Gladstone EJ, Rutherford K, Morgan SG. Patterns and trends in long-term opioid use for non-cancer pain in British Columbia, 2005–2012. Can J Public Health. 2016 Jul;107(4–5):e404–9.

12. Smolina K, Crabtree A, Chong M, Zhao B, Park M, Mill C, et al. Patterns and history of prescription drug use among opioid-related drug overdose cases in British Columbia, Canada, 2015–2016. Drug and Alcohol Dependence. 2019 Jan 1;194:151–8.

13. Oliva EM, Bowe T, Manhapra A, Kertesz S, Hah JM, Henderson P, et al. Associations between stopping prescriptions for opioids, length of opioid treatment, and overdose or suicide deaths in US veterans: observational evaluation. BMJ. 2020 Mar 4;m283.

14. Smolina K, Crabtree A, Chong M, Park M, Mill C, Zhao B, et al. Prescription-related risk factors for opioid-related overdoses in the era of fentanyl contamination of illicit drug supply: A retrospective case-control study. Substance Abuse. 2020 May 22;0(0):1–7.

15. Janjua NZ, Islam N, Kuo M, Yu A, Wong S, Butt ZA, et al. Identifying injection drug use and estimating population size of people who inject drugs using healthcare administrative datasets. International Journal of Drug Policy. 2018 May 1;55:31–9.

16. Quan H, Sundararajan V, Halfon P, Fong A, Burnand B, Luthi J-C, et al. Coding Algorithms for Defining Comorbidities in ICD-9-CM and ICD-10 Administrative Data. Medical Care. 2005 Nov;43(11):1130–9.
