# Supplementary material for: Discontinuation and tapering of prescribed opioids and risk of overdose among people on long-term opioid therapy for pain with and without opioid use disorder in British Columbia, Canada: A retrospective cohort study
Source: PLoS Med. 2022 Dec 1;19(12):e1004123. doi: 10.1371/journal.pmed.1004123 (PMC9714711; doi:10.1371/journal.pmed.1004123)
Supplement: S2 Text — (DOC) [file pmed.1004123.s003.doc]

**Data Analysis Plan**

**Date of request**: October 18, 2020

**Name(s)**: Mary Clare Kennedy, Lianping Ti, Alexis Crabtree, Seonaid Nolan, Wing Yin Mok, Zishan Cui, Mei Chong, Amanda Slaunwhite.

**Priority (indicate high upon PI’s approval)**: Normal

**Cohort(s)**: BC Provincial Overdose Cohort - Reference Cohort (as defined in [1]) without overdose cases removed.

**Study Title**: Discontinuation of prescription opioid treatment for pain and risk of overdose among people with and without opioid use disorder.

Study Objective(s): Drawing on data from individuals on chronic opioid therapy for pain in the Reference Cohort, we will measure the association between discontinuation/tapering of prescription opioid treatment for pain and risk of overdose, stratified by opioid use disorder and prescribed opioid agonist treatment (OAT) status.

Study Period: October 3, 2014 to December 31, 2018.

Note: The above refers to the main study period. However, we will also define the effect modifier variable and some covariates based on data from up to 3 years prior to participant follow-up start dates.

Study Sample (include inclusion and exclusion criteria):

Inclusion criteria:

All participants in the Reference Cohort who have one or more episodes of long-term (≥90 days), chronic (≥90% of those 90 days on therapy) use of prescription opioids for pain treatment (see definition of opioids for pain below) between October 3, 2014 and June 30, 2018.

Note: October 3, 2014 is the beginning of the study period so that the earliest possible follow-up start date (i.e., day 91 of a treatment episode, as defined below) is January 1, 2015 (the date when overdose events began to be recorded with additional sources).

-Follow-up start date = 1^st^ day after the first eligible episode within the above-noted study period.

-Follow-up end date = death date or end of study (i.e., December 31, 2018), whichever occurs first.

-Participants must be present in the client roster at least 3 years prior to the follow-up start date and all subsequent years until the year of follow-up ended.

-Opioids for pain definition: Based on prescribing identified via DIN/PIN and includes all opioids listed in Appendix 1, except for OAT (i.e., methadone and buprenorphine/naloxone for opioid use disorder specifically and Kadian for opioid use disorder after June 5, 2017, as noted below), as identified in the dataset through pharmacy fee codes that identify the use of medications for opioid use disorder specifically). All other opioids (including methadone and buprenorphine for use other than as OAT) were classified as opioids for pain [2].

Slow-release oral morphine used as OAT prior to June 5, 2017 could not be assessed due to the absence of OAT fee codes for this medication prior to this time point; however, its use for opioid use disorder was extremely uncommon in BC until 2017. Beginning on June 5, 2017, Kadian with an OAT PIN is defined as OAT for this study and should therefore be excluded from the definition of opioids for pain from this point onward in the study period.

Exclusion criteria:

1) Age at follow-up start date <14 or >74.

2) Diagnosed with cancer/palliative care at any time point during the study period. This refers to participants who had at least one hospitalization or two physician billings no more than a year apart in which they had a cancer/palliative care diagnosis. Cancer diagnosis is defined as (1) all malignant neoplasms; (2) neoplasms of uncertain behaviour of unspecified nature; (3) carcinomas in situ; and (4) other unspecified malignant neoplasms of skin. The respective ICD-10 codes for these categories are (1) C00-C42 and C45-C97; (2) D37-D48; (3) D00-D02 and D04-D09; (4) C44.  This includes all codes under neoplasms in the following link except for benign neoplasms (D10-D36) and melanoma-related codes (C43 and D03):  [https://icd.who.int/browse10/2016/en#!/II](https://icd.who.int/browse10/2016/en#!/II)

The ICD-10 for palliative care encounters is Z51.5.

3) Experienced overdose (as defined below) between day 1 and day 90 of an eligible long-term chronic PO treatment episode (i.e., before day 1 of follow-up) unless that person has a subsequent eligible episode of long-term chronic use of POs for pain (as defined above) and does not experience an opioid-related overdose before the follow-up start date (i.e., between day 1 and day 90) of that subsequent treatment episode.

Censoring:

-Death.

-At December 31, 2018 (end of study period).

Main outcome measure(s):

Overdose, both non-fatal and fatal (yes vs. no), between 2015 and 2018, defined as follows (based on Provincial Overdose Cohort Technical Notes):

*Non-fatal overdose:*

Patient Care Information System:

-Naloxone administered by paramedics OR

-Impression code: Recreational Drug OD AND CARD in (9,23,26,31)*, OR

-Impression code: Cardiac arrested: treated and CARD 23

Siren

-Naloxone administered, OR

-Impression code: ‘Opioid-related’, ‘opioid-related/OD’, OR

- Impression: Cardiac arrest and CARD 23*

DPIC

-Any call with a code related to drug overdose/ poisoning.

Enhanced ED

-Case-based reported by Emergency Departments by participating health authority

MSP, DAD, NACRS

-Case defined by ICD version 9 and 10 (e.g., 965.0- poisoning by opiates and related narcotics; E850,0- accidental poisoning by opiates and related narcotics)

*CARD 9: Cardiac or respiratory arrest/death; CARD 23: Overdose/poisoning (ingestion); CARD 26: Sick; CARD 31: Unconscious.

*Fatal overdose:*

BCCS:

-open investigations (toxicology pending) and closed drug overdose deaths

Other:

-Vital statistics deaths

-Deaths captured through the drug-related overdose algorithm (i.e., if death date from other administrative databases (e.g., client roster) lies between the start date and end date of an overdose episode, then this overdose episode is defined as a fatal overdose. In these cases, please use the overdose episode start date as the date of the fatal overdose.).

*Note: Related events present in multiple data sets and occurring within 24 hours of each other should be grouped into a single overdose episode to prevent double counting of overdoses.*

Main exposure measure:

Summary definition:

Prescription opioid discontinuation/tapering (3-level measure), based on days that supply was dispensed:

(1) Continued therapy (<7-day gap(s) in therapy (following the date at which the supply of a prescription would have run out if taken every day));

(2) Tapering (≥2 sequential decreases in dose of ≥5 morphine milligram equivalents, where such dose decreases are separated by ≤42 days; defined in detail below);

(3) Discontinued (not received opioids ≥7 days (following the date at which the supply of a prescription would have run out if taken every day).

Detailed definition of main exposure measure (2 stages):

STAGE 1:

**(A)** Identify opioid therapy treatment episodes- defined based on a group of one or more dispensations with less than 182-day gap from the last day of supply of the previous dispensation to the next dispensation date [3,4]. That is, episodes are separated by at least 182 days. Therefore, an individual can have multiple episodes during their follow-up period if their follow-up period is long enough to have multiple episodes. Episodes can be either acute or long-term. However, only **long-term** (≥90 days) **chronic** (≥90% days on therapy) episodes should be used for **tapering** assessment.

**(B)** Each episode of being on opioid therapy is partitioned every **14 days** into a tapering period (TP) until the end of the episode.

**(C)** Calculate the **Average daily dose in each TP** (calculated as the total of daily morphine milligram equivalents (ME)/total days on therapy in each TP (i.e., maximum denominator would be 14 days)).

***Note:*** *Definition of dose/ME provided in Appendix 2.*


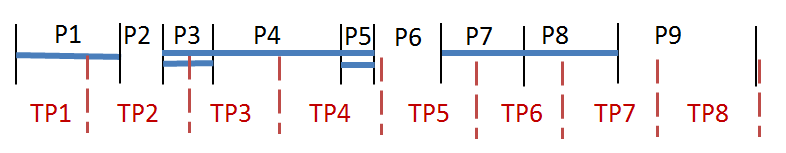


***Note:*** *P1, P2…PX are defined by changes in prescriptions (i.e., P1 = first prescription; P2 = no prescription; P3 = begins when second and third prescriptions begin and ends when third prescription ends; P4 = begins at end of third prescription and ends when fourth prescription begins etc.)*

**To calculate average daily ME during TP2**:

1. (# of days from P1 that fall into TP2*average daily ME from P1) + (# of days from P3 that fall into TP2*average daily ME from P3) 🡪 this gives **total MEs** during TP2.
2. To get the **average daily MEs** during TP2, take the **total MEs** calculated in Step 1 and **divide by total number of days on therapy** (i.e., days with an active Rx from P1 & P3 that fall into TP2).

**(D)** Calculate the relative ***change* in daily average ME** **between consecutive TPs** to categorize remaining TPs into continued/discontinued/ tapering (tapering definition below).

If a gap between **2 consecutive tapering TPs** is ≥14 days, then further divide into 2 separate tapering episodes.

**Tapering definition (for Stage 1):**

When the following **three criteria** are met, **tapering starts** at the beginning of TP(t) with the first decrease in average daily ME and continues until tapering ends:

- - 1. There are at least two relative decreases of ≥5% in the average daily ME from one TP to subsequent TPs AND
  - 2. Each dose decrease is separated by ≤3 TPs (i.e., 42 days) AND
  - 3. The average dose in the TP after the second decrease is < the average dose between the two decreases < the average dose in the TP before the first decrease.

**Note:** Individuals should be defined as tapering even if the decreases of ≥5% in the average daily ME are not in consecutive TPs but each decrease occurs within 42 days (i.e., ≤3 TPs).

- **Tapering ends** at the last day on therapy in TP(t) if one of the following scenarios is met:
  - There is a break in treatment of 14+ days from the last day of treatment in TP(t) and the beginning of treatment in TP(t+1).
  - No subsequent decrease of ≥5% in average daily ME for >3TPs.
  - TP(t+2) ≥ TP(t+1) > TP(t) (i.e., average daily ME continues to increase for 2 consecutive TPs or increases for immediately next TP and then remains stable in next TP after that).
  - TP(t+1) > TP(t+2) > TP(t) (i.e., increase in average daily ME but then decrease to a dose that is still higher than the dose before it was increased).

**Stage 2** (to categorize each day within an episode as continued, tapering or discontinued therapy)**:**

For each continued or tapering TP (i.e., non-discontinued TPs) defined in Stage 1, if there is a ≥7-day gap in treatment within one of these TPs or between 2 consecutive non-discontinued TPs, this gap should be categorized as discontinued. The rest of the days within a single TP should retain the same TP categorization.

If a <7-day gap lies between a ‘tapering’ TP and a ‘continued’ TP, then that gap should be categorized as the treatment status prior to the gap. That is, if the ‘continued’ TP occurred before the ‘tapering’ TP then the gap between these statuses is to be categorized as continued. However, if the ‘tapering’ TP occurred before the ‘continued’ TP, the gap is to be categorized as tapering.

Effect modifier measure:

**Opioid use disorder (OUD) and prescribed opioid agonist therapy (OAT) status**, three-level, time-updated, measured based on Medical Services Plan (MSP), Discharge Abstract Database (DAD) and Pharmanet (DIN/PIN) data:

1. No OUD diagnosis in the past 3 years;
2. OUD diagnosis in the past 3 years but not prescribed OAT in the past 3 years*;
3. OUD diagnosis in the past 3 years and prescribed OAT in the past 3 years*.

***Update- July 4, 2022:** In response to a reviewer’s comment, we should instead use the following 3-level measure (using past-90-day rather than past-3-year OAT prescription) to aid us in identifying the role of more recent OAT prescription in modifying the associations of interest:

1. No OUD diagnosis in the past 3 years;
2. OUD diagnosis in the past 3 years but not prescribed OAT in the **past 90 days;**
3. OUD diagnosis in the past 3 years and prescribed OAT in the **past 90 days.**

**Definitions of OUD diagnosis and OAT should be measured as follows:**

| **Diagnosis** | **ICD9 codes** | **ICD10 codes** | **Definition** | **Datasets** |
| --- | --- | --- | --- | --- |
| Opioid use disorder | 304.00 – 304.03  304.70 – 304.73 305.50-305.53 | F11 | Either 2 primary care visits or 1 hospitalization for OUD in a given year in the past 3 years | Medical Services Plan (MSP); Discharge Abstract Database (DAD) |

**Opioid use disorder**

**Medications included in OAT category**

| **Medication group** | **Medications included** | **Dataset** |
| --- | --- | --- |
| Opioid agonist therapy | Buprenorphine (for opioid use disorder), methadone (for opioid use disorder), or slow-release oral morphine (for opioid use disorder) prescribed at least once in the past 90 days | PharmaNet (DIN/PIN)* |

***Note:** DIN/PIN for OAT are specifically allocated for OUD treatment (as opposed to the treatment of pain).

Preliminary statistical analyses (to be conducted prior to primary/secondary analyses):

-To develop a study sample flow chart, please provide frequencies (n/%) of (a) the Reference Cohort (for step 1, below) or (b) the sample from the immediately next step (steps 2 through 4, below) who were excluded from the study sample for each of the following reasons:

1. Did not have at least one long-term, chronic prescription opioid treatment episode (as defined above) between October 3, 2014 and June 30, 2018.

2. Overdose occurred prior to day 91 (follow-up start date) of qualifying opioid treatment episode and do not have a subsequent eligible long-term, chronic opioid treatment episode during the study period where they don’t experience an overdose prior to the follow-up start date of that subsequent treatment episode).

*3.* Out of age range at day 1 (14-74 years).

4. Cancer/palliative diagnosis at any point during the study period.

-Please provide frequencies (n/%) of the study sample who experienced:

1. at least one overdose (either non-fatal or fatal) during follow-up.

1a. Among those who have experienced at least one overdose (either non-fatal or fatal) event during follow-up, please provide the breakdown of the number of overdose events experienced during follow-up (i.e., the n/% of these individuals who experienced 1 event, 2 events, 3 events etc.).

2. at least one non-fatal overdose event during follow-up.

2a. Among those who have experienced at least one non-fatal overdose event during follow-up,

please provide the breakdown of the number of non-fatal overdose events experienced during

follow-up (i.e., the n/% of these individuals who experienced 1 event, 2 events, 3 events etc.).

3. a fatal overdose event during follow-up.

Note: Please provide all of the aforementioned overdose event frequencies for the overall sample and stratified by OUD and OAT status.

-Please provide frequencies (n/%) for the full study sample and stratified by OUD/ OAT status who experienced:

1. at least one PO discontinuation event during follow-up,

1a. Among those who have experienced at least one PO discontinuation event during follow-up, please provide the breakdown of the number of discontinuation events experienced during follow-up (i.e., the n/% of these individuals who experienced 1 event, 2 events, 3 events etc.) as well as the median duration (and Q1 and Q3 of the interquartile range [IQR]) of discontinuation periods.

2. at least one PO tapering event during follow-up.

2a. Among those who have experienced at least one PO tapering event during follow-up, please provide the breakdown of the number of tapering events experienced during follow-up (i.e., the n/% of these individuals who experienced 1 event, 2 events, 3 events etc.) as well as the median duration (and Q1 and Q3 of the IQR) of tapering periods.

Primary statistical analyses*- STEP ONE*

- Please provide the total observation time contributed by the study sample, as well as the median and Q1/Q3 of the IQR, and minimum and maximum of the range of observation time per participant.

-Please use the person-time method to calculate the incidence density rate of (overdose (either non-fatal or fatal); (2) non-fatal overdose; (3) fatal overdose, overall and stratified by OUD/OAT status.

-As a first step for survival analyses, please use unadjusted Cox regression analyses for recurrent events with time-updated covariates to calculate the unadjusted hazard ratio for the association between PO discontinuation/tapering (reference group= continuous PO therapy) with time to overdose, stratified by OUD/OAT status (i.e., fit separate unadjusted models for each of the three levels of OUD/OAT status).

-For these Cox analyses, please treat prescription opioid discontinuation/tapering as a time-updated variable based on current exposure status (i.e., currently in period of discontinuation, tapering, or continuous treatment).

-Please provide basic descriptive statistics (i.e., frequencies (n/%) for categorical variables & median and Q1/Q3 of the IQR, minimum and maximum of the range for continuous variables) for the baseline characteristics (variables shown in Table 1 below) of participants, stratified by OUD/OAT status at baseline. Use Pearson’s Chi-square test (or Fisher’s exact test when expected cell counts are <5) to compare categorical explanatory variables and the Kruskal Wallis test to compare continuous variables.

Table 1- Potential confounding variables

| **Variable** | **Variable name/ definition** | **Comparisons** |
| --- | --- | --- |
| Age at day 1 of follow-up (FU) | age_at_day1 | Per year older |
| Sex | gender | Female *vs.* Male |
| Geographic location at day 1 of FU | LHA_at_day1 | Vancouver; Fraser; Island; Interior; Northern. |
| Calendar year | fup_start_year | 2015; 2016; 2017; 2018. |
| Average daily ME** | Definition in Appendix 2 | 0-49 ME/day; 50-89 ME/day; 90-199 ME/day; 200+ ME/day. |
| Type of opioid treatment** | Definition in Appendix 2 (hierarchal measure) | Long acting opioid, short acting opioid vs. tramadol only |
| Elixhauser index score (without mental health conditions)* | Definition in Appendix 3 | 0 vs. 1 vs. 2+ |
| Respiratory comorbidities* | Definition in Appendix 3 | Yes *vs.* No |
| Cardiovascular comorbidities* | Definition in Appendix 3 | Yes *vs.* No |
| Mental health conditions* ^ | Definition in Appendix 3 | Yes *vs.* No |
| Injection drug use* | Definition in Appendix 3 | Yes *vs.* No |
| Benzodiazepines/z-drugs** | Definition in Appendix 3 | Yes *vs.* No |
| Other sedating medications** | Definition in Appendix 3 | Yes *vs.* No |
| Non-sedating antidepressants** | Definition in Appendix 3 | Yes *vs.* No |
| Non-sedating antipsychotics** | Definition in Appendix 3 | Yes *vs.* No |
| Hospitalization*** | Definition in Appendix 3 | Yes *vs.* No |
| Incarceration*** | Definition in Appendix 3 | Yes *vs.* No |
| **Notes:**  *Assessed during three years prior to day 1 of FU.  **Assessed during the 90 days prior to day 1 of FU.  ***Assessed during the 30 days prior to day 1 of FU.  ^ If a participant meets the definition of having both a ‘less severe’ and ‘more severe’ mental health condition, they would be defined as having a more severe mental health condition. | | |

Please use unadjusted Cox regression analyses for recurrent events (with time-update covariates) to calculate unadjusted hazard ratios for the association between each variable listed in the above table with time to overdose (either non-fatal or fatal), fitting separate models for each level of OUD/OAT status.

After reviewing the bivariable Cox regression results, we will proceed with building multivariable models.

**UPDATE- MARCH 15, 2021:**

To estimate the effect of the main exposure of PO treatment status (i.e., discontinued treatment, tapered treatment vs. continued therapy) on risk of non-fatal/fatal overdose (the primary outcome of interest), please use marginal structural modelling with inverse probability of treatment weights (IPTWs) given that this method can handle time-dependent variables that are both confounders of the effect of interest and predicted by previous PO treatment status, and can also adjust for selection bias [5]. Please fit separate models for each of the 3 levels of OUD/OAT status. Please use stabilized weights given that unstable weights can potentially lead to estimators with large variances [5]. All confounding variables listed in Table 1 above (but treated as time-updated) will be considered for calculating the weights.

To calculate stabilized IPTWs, first calculate the denominator, which is the probability of a participant being in each level of the exposure (i.e., discontinuation, tapering) given their past history of exposure and prognostic factor history (i.e., confounding variables listed above). Then, calculate the numerator, which is the probability of a participant being in each level of the exposure conditional on their past history of exposure and baseline covariates. Finally, please use marginal structural Cox regression to estimate the effect of PO treatment status on overdose after adjusting for the stabilized weights calculated.

**Secondary statistical analyses**:

**UPDATE- JULY 14, 2022 (in response to PLoS Medicine reviewers’ comments):**

1. Please re-run the aforementioned marginal structural Cox regression analyses but as a series of models that are increasingly adjusted for groups of confounders, starting with demographic variables (age; sex; regional health authority; calendar year), followed by prescription and drug-related variables (average daily MME; prescribed opioid type; injection drug use; benzodiazepine/z-drug use; other sedating medication use; non-sedating anti-depressant use; non-sedating antipsychotic use), then comorbidity variables (Elixhauser index score (without mental health conditions); respiratory comorbidities; cardiovascular comorbidities; mental health conditions), and finally institutionalization variables (hospitalization; incarceration).
2. As a sensitivity analysis, please re-run the bivariable and marginal structural Cox regression analyses using an alternative measure of the exposure of interest (prescribed opioid for pain treatment status), where continued and discontinued therapy were defined as <14-day and ≥14-day gap(s), respectively, in therapy following the date at which the supply of a prescription would have run out if taken every day.
3. Please provide the rate of transitions (per 100 person-years) between levels of diagnosed OUD and prescribed OAT status among the study sample during follow-up.

**Appendix 1:**

**Medications included in opioid class:**

| anileridine |
| --- |
| buprenorphine |
| butorphanol |
| codeine |
| diamorphine |
| fentanyl |
| hydrocodone |
| hydromorphone |
| levorphanol |
| meperidine |
| methadone |
| morphine |
| oxycodone |
| oxymorphone |
| pentazocine |
| propoxyphene |
| tapentadol |
| tramadol |

**Appendix 2:**

**Opioid dose definition:**

Measured in morphine milligram equivalents (ME), based on the following formula (from [6]):

ME units per day = strength per unit (number of units/day) x ME conversion factor.

Example: “An individual using one oxycodone 40mg tablet orally in the morning and one 40mg tablet at night: 40mg x 2 doses per day x conversion factor of 1.5=120mg OME per day” [6].

Information regarding ME conversion factors for specific medications can be found in Nielsen et al. 2016 [6].

**To calculate average daily ME in most recent opioid treatment episode (in episodes when there are multiple active prescriptions (3 prescriptions in the example below)**:

1. (# of days from prescription 1 (P1) that fall into the treatment episode*average daily ME from P1) + (# of days from P2 that fall into the treatment episode*average daily ME from P2) + (# of days from P3 that fall into the treatment episode*average daily ME from P3) 🡪 this gives **total MEs** during the treatment episode.
2. To get the **average daily MEs** during the treatment episode, take the total MEs calculated in 1 and divide by total number of days on therapy (i.e., days with an active Rx from P1, P2, P3 that fall into the treatment episode).

**Type of opioid treatment:** Refers to opioid prescribed in previous treatment episode, with a **hierarchal classification** as follows: long acting, short acting opioid, or tramadol only [7].

**Appendix 3 (Sociodemographic, physical health, mental health and non-opioid prescription potential confounding variable measures):**

Elixhauser comorbidity index score (without mental health conditions): derived from DAD and modified by excluding ICD10 codes for mental health conditions [2,8]. Defined the same as detailed at the following link but without mental heath conditions included:

<http://mchp-appserv.cpe.umanitoba.ca/viewConcept.php?conceptID=1436>

Physical health diagnoses (respiratory comorbidities; cardiovascular comorbidities) are based on case definitions used by the BC Ministry of Health [9].

-Respiratory comorbidities: includes Asthma and Chronic Obstructive Pulmonary Disease.

-Cardiovascular comorbidities: includes ischemic heart disease, heart failure, hypertension, stroke.

***ICD9/10 codes used to derive mental health conditions variable (hierarchal measure where if a person has conditions classified as both more and less severe, that person would be defined as having more a severe condition):***

| **Variable** | **Comparisons** | **ICD9 codes** | **ICD10 codes** | **Definition** | **Datasets** |
| --- | --- | --- | --- | --- | --- |
| Mental health Conditions | More severe, less severe vs. no mental health condition | **More severe:**  Schizophrenia (295, 297, 298);  Bipolar disorder (296);  Personality disorder (301).  **Less severe:**  Depression (300.4; 311; 50B (also requires another code to qualify));  Anxiety (300 (excluding 300.4); 50B (also required another code to qualify));  Stress/ adjustment disorders (308; 309). | **More severe:**  Schizophrenia (F20, F21, F22, F23, F24, F25, F28, F29);  Bipolar disorder (F30, F31, F34 (excluding F34.1), F38, F39);  Personality disorder (F60, F61, F69).  **Less severe:**  Depression (F32, F33, F34.1);  Anxiety (F40, F41));  Stress/ adjustment disorders (F43). | Either 2 primary care visits or 1 hospitalization in a given year in the past 3 years | MSP, DAD |

Injection drug use: flagged as being a person who used intravenous drugs using a validated algorithm that requires diagnostic codes for substance use as well as injection-related infections [10].

Non-opioid for pain prescription measures, as identified in the dataset through pharmacy fee codes (Benzodiazepines/z-drugs; Other sedating medications; Non-sedating antidepressants; Non-sedating antipsychotics).

***Benzodiazepines and z-drugs***

| alprazolam |
| --- |
| bromazepam |
| chlordiazepoxide |
| clobazam |
| clonazepam |
| clorazepate |
| diazepam |
| estazolam |
| flurazepam |
| ketazolam |
| lorazepam |
| nitrazepam |
| oxazepam |
| temazepam |
| triazolam |
| zaleplon |
| zolpidem |
| zopiclone |

**Other sedating medications:**

Amitriptyline, amoxapine, clomipramine, desipramine, doxepin, imipramine, maprotiline, mirtazapine, nefazodone, nortriptyline, phenelzine, protriptyline,tranylcypromine, trazodone, trimipramine, tryptophan, asenapine, butaperazine, chlorpromazine, chlorprothixene, clozapine, flupentixol, fluspirilene, loxapine, mesoridazine, methotrimeprazine, olanzapine, periciazine, piperacetazine, pipotiazine, prochlorperazine, promazine, quetiapine,

remoxipride, risperidone, thiopropazate, thioproperazine, thioridazine, ziprasidone, zuclopenthixol, gabapentin,

pregabalin, carbamazepine, lamotrigine, levetiracetam, phenytoin, topiramate, valproic acid, baclofen, cyclobenzaprine, methocarbamol, choral hydrate, phenobarbital, nabilone

Non-sedating antidepressants

| bupropion |
| --- |
| citalopram |
| desvenlafaxine |
| duloxetine |
| escitalopram |
| fluoxetine |
| fluvoxamine |
| isocarboxazid |
| levomilnacipran |
| moclobemide |
| nomifensine |
| paroxetine |
| sertraline |
| venlafaxine |
| vilazodone |
| vortioxetine |

Non-sedating antipsychotics

| aripiprazole |
| --- |
| fluphenazine |
| haloperidol |
| lurasidone |
| paliperidone |
| perphenazine |
| pimozide |
| tiotixene |
| trifluoperazine |

Hospitalization: based on DAD data and defined as at least one hospital admission in last 30 days.

Incarceration: based on BC Correction Services Data and defined as at least one incarceration event in last 30 days.

**Reference:**

1. MacDougall L, Smolina K, Otterstatter M, Zhao B, Chong M, Godfrey D, et al. Development and characteristics of the Provincial Overdose Cohort in British Columbia, Canada. Lima VD, editor. PLoS ONE. 2019 Jan 10;14(1):e0210129.

2. Smolina K, Crabtree A, Chong M, Park M, Mill C, Zhao B, et al. Prescription-related risk factors for opioid-related overdoses in the era of fentanyl contamination of illicit drug supply: A retrospective case-control study. Substance Abuse. 2020 May 22;0(0):1–7.

3. Smolina K, Gladstone EJ, Rutherford K, Morgan SG. Patterns and trends in long-term opioid use for non-cancer pain in British Columbia, 2005–2012. Can J Public Health. 2016 Jul;107(4–5):e404–9.

4. Smolina K, Crabtree A, Chong M, Zhao B, Park M, Mill C, et al. Patterns and history of prescription drug use among opioid-related drug overdose cases in British Columbia, Canada, 2015–2016. Drug and Alcohol Dependence. 2019 Jan 1;194:151–8.

5. Pazzagli L, Linder M, Zhang M, Vago E, Stang P, Myers D, et al. Methods for time-varying exposure related problems in pharmacoepidemiology: An overview. Pharmacoepidemiol Drug Saf. 2018 Feb;27(2):148–60.

6. Nielsen S, Degenhardt L, Hoban B, Gisev N. A synthesis of oral morphine equivalents (OME) for opioid utilisation studies: Oral Morphine Equivalents. Pharmacoepidemiol Drug Saf. 2016 Jun;25(6):733–7.

7. Oliva EM, Bowe T, Manhapra A, Kertesz S, Hah JM, Henderson P, et al. Associations between stopping prescriptions for opioids, length of opioid treatment, and overdose or suicide deaths in US veterans: observational evaluation. BMJ. 2020 Mar 4;m283.

8. Quan H, Sundararajan V, Halfon P, Fong A, Burnand B, Luthi JC, et al. Coding Algorithms for Defining Comorbidities in ICD-9-CM and ICD-10 Administrative Data. Medical Care. 2005 Nov;43(11):1130–9.

9. British Columbia Centre for Disease Control: Chronic Disease Dashboard Case Definitions [Internet]. 2020 [cited 2020 Jun 18]. Available from: http://www.bccdc.ca/health-info/disease-system-statistics/chronic-disease-dashboard#Case--Definitions

10. Janjua NZ, Islam N, Kuo M, Yu A, Wong S, Butt ZA, et al. Identifying injection drug use and estimating population size of people who inject drugs using healthcare administrative datasets. International Journal of Drug Policy. 2018 May 1;55:31–9.
