# Supplementary material for: Discontinuation and tapering of prescribed opioids and risk of overdose among people on long-term opioid therapy for pain with and without opioid use disorder in British Columbia, Canada: A retrospective cohort study
Source: PLoS Med. 2022 Dec 1;19(12):e1004123. doi: 10.1371/journal.pmed.1004123 (PMC9714711; doi:10.1371/journal.pmed.1004123)
Supplement: S3 Text — (DOCX) [file pmed.1004123.s004.docx]

**S3 Text: Supplementary analyses**

Table A. Transition rate (per 100 person-years) between levels of diagnosed opioid use disorder (OUD) and prescribed opioid agonist therapy (OAT) status during follow-up among people on long-term opioid therapy for pain in British Columbia, Canada *n* = 14,037).

|  | **To** | | |
| --- | --- | --- | --- |
| **From** | **No diagnosed opioid use disorder (OUD)**‡ | **Diagnosed OUD**‡ **but not prescribed opioid agonist therapy (OAT)**† | **Diagnosed OUD**‡ **and prescribed OAT**† |
| **No diagnosed opioid use disorder (OUD)**‡ | - | 0.8 | 0.1 |
| **Diagnosed OUD**‡ **but not prescribed opioid agonist therapy (OAT)**† | 29.5 | - | 0.4 |
| **Diagnosed OUD**‡ **and prescribed OAT**† | 25.9 | 11.9 | - |
| ‡ Refers to past three years.  † Refers to past 90 days. | | | |

**Table B. Regression analyses for the effects of discontinuation and tapering of prescribed opioid treatment for pain on risk of overdose among people on long-term opioid therapy for pain in British Columbia, Canada, stratified by diagnosed opioid use disorder (OUD) and prescribed opioid agonist therapy (OAT) status.**

|  | **Unadjusted estimates** | **Adjusted estimates^1^** | **Adjusted estimates^2^** | **Adjusted estimates^3^** | **Fully adjusted estimates^4^** |
| --- | --- | --- | --- | --- | --- |
|  | **Hazard ratio**  **(95% CI**) | **Hazard ratio**  **(95% CI**) | **Hazard ratio**  **(95% CI**) | **Hazard ratio**  **(95% CI**) | **Hazard ratio**  **(95% CI**) |
| **No diagnosed opioid use disorder (OUD)**‡ |  |  |  |  |  |
| Continued prescribed opioid treatment | Ref | Ref | Ref | Ref | Ref |
| Tapered prescribed opioid treatment | 1.59 (1.19, 2.13) | 1.16 (0.86, 1.55) | 1.14 (0.84, 1.53) | 1.14 (0.84, 1.53) | 1.14 (0.84, 1.53) |
| Discontinued prescribed opioid treatment | 1.24 (0.98, 1.57) | 1.46 (1.15, 1.84) | 1.44 (1.13, 1.84) | 1.44 (1.13, 1.85) | 1.44 (1.12, 1.83) |
| **Diagnosed OUD**‡ **but not prescribed opioid agonist therapy (OAT)**† |  |  |  |  |  |
| Continued prescribed opioid treatment | Ref | Ref | Ref | Ref | Ref |
| Tapered prescribed opioid treatment | 0.47 (0.22, 1.01) | 0.34 (0.16, 0.74) | 0.31 (0.14, 0.67) | 0.31 (0.14, 0.67) | 0.31 (0.14, 0.67) |
| Discontinued prescribed opioid treatment | 2.34 (1.36, 4.04) | 2.98 (1.72, 5.14) | 3.07 (1.80, 5.22) | 3.06 (1.80, 5.19) | 3.18 (1.87, 5.40) |
| **Diagnosed OUD**‡ **and prescribed OAT**† |  |  |  |  |  |
| Continued prescribed opioid treatment | Ref | Ref | Ref | Ref | Ref |
| Tapered prescribed opioid treatment | 0.75 (0.39, 1.47) | 0.62 (0.32, 1.20) | 0.67 (0.33, 1.33) | 0.66 (0.33, 1.31) | 0.61 (0.30, 1.22) |
| Discontinued prescribed opioid treatment | 0.95 (0.63, 3.86) | 2.83 (1.91, 4.20) | 2.59 (1.74, 3.85) | 2.54 (1.71, 3.78) | 2.52 (1.68, 3.78) |
| CI = confidence interval; OUD = opioid use disorder; OAT = opioid agonist therapy.  ^1.^ Using marginal structural modelling with inverse probability of treatment weights (IPTW), models adjusted for sociodemographic variables (sex; age; regional health authority; calendar year).  ^2.^ Using marginal structural modelling with IPTW, models adjusted for sociodemographic variables and prescription and drug-related variables (average daily morphine milligram equivalents (MME); type of prescribed opioid; cumulative days in opioid treatment during follow-up; injection drug use; benzodiazepine/z-drug use; other sedating medication use; non-sedating antidepressant use; non-sedating antipsychotic use).  ^3.^ Using marginal structural modelling with IPTW, models adjusted for sociodemographic variables; prescription and drug-related variables; and comorbidity variables (Elixhauser index score (without mental health conditions); respiratory comorbidities; cardiovascular comorbidities; mental health conditions).  ^4.^ Fully adjusted models using marginal structural modelling with IPTW - adjusted for sociodemographic variables; prescription and drug-related variables; comorbidity variables; and institutionalization variables (hospitalization; and incarceration).  ‡ Refers to past three years.  † Refers to past 90 days. | | | | | |

**Table C. Sensitivity analyses using an alternative measure of treatment discontinuation (defined ≥14 day-gap(s) in therapy) in marginal structural models estimating the effects of discontinuation and tapering of prescribed opioid treatment for pain on risk of overdose among people on long-term opioid therapy for pain in British Columbia, Canada, stratified by diagnosed opioid use disorder (OUD) and prescribed opioid agonist therapy (OAT) status.**

|  | **Adjusted estimates*** | |
| --- | --- | --- |
|  | **Hazard ratio**  **(95% CI**) | ***p-*value** |
| **No diagnosed opioid use disorder (OUD)**‡ |  |  |
| Continued prescribed opioid treatment | Ref |  |
| Tapered prescribed opioid treatment | 1.12 (0.83, 1.50) | 0.465 |
| Discontinued prescribed opioid treatment | 1.38 (1.08, 1.77) | 0.010 |
| **Diagnosed OUD**‡ **but not prescribed opioid agonist therapy (OAT)**† |  |  |
| Continued prescribed opioid treatment | Ref |  |
| Tapered prescribed opioid treatment | 0.31 (0.14, 0.67) | 0.003 |
| Discontinued prescribed opioid treatment | 3.15 (1.86, 5.35) | <0.001 |
| **Diagnosed OUD**‡ **and prescribed OAT**† |  |  |
| Continued prescribed opioid treatment | Ref |  |
| Tapered prescribed opioid treatment | 0.60 (0.30, 1.21) | 0.153 |
| Discontinued prescribed opioid treatment | 2.47 (1.65, 3.69) | <0.001 |
| CI = confidence interval; OUD = opioid use disorder; OAT = opioid agonist therapy.  *Using marginal structural modelling with inverse probability of treatment weights (IPTW), models adjusted for: calendar year; sex; age; regional health authority; average daily morphine milligram equivalents (MME); type of prescribed opioid; Elixhauser index score (without mental health conditions); respiratory comorbidities; cardiovascular comorbidities; mental health conditions; injection drug use; benzodiazepine/z-drug use; other sedating medication use; non-sedating antidepressant use; non-sedating antipsychotic use; hospitalization; and incarceration.  ‡ Refers to past three years.  † Refers to past 90 days. | | |
